# Older adults with acquired brain injury: a population based study

**DOI:** 10.1186/1471-2318-13-97

**Published:** 2013-09-23

**Authors:** Vincy Chan, Brandon Zagorski, Daria Parsons, Angela Colantonio

**Affiliations:** 1 Toronto Rehabilitation Institute, University Health Network, 550 University Avenue, Toronto, Ontario, M5G 2A2, Canada; 2 Graduate Department of Rehabilitation Science, University of Toronto, 500 University Avenue, Toronto, Ontario M5G 1V7, Canada; 3 Institute of Health Policy, Management, and Evaluation, University of Toronto, 155 College Street, Toronto, Ontario M5T 3M6, Canada; 4 Ontario Neurotrauma Foundation, 90 Eglinton Avenue East, Suite 601, Toronto, Ontario M4P 2Y3, Canada; 5 Department of Occupational Science & Occupational Therapy, University of Toronto, 500 University Avenue, Toronto, Ontario M5G 1V7, Canada

**Keywords:** Brain injury, Epidemiology, Outcomes

## Abstract

**Background:**

Acquired brain injury (ABI), which includes traumatic (TBI) and non-traumatic brain injury (nTBI), is a leading cause of death and disability worldwide. The objective of this study was to examine the trends, characteristics, cause of brain injury, and discharge destination of hospitalized older adults aged 65 years and older with an ABI diagnosis in a population with universal access to hospital care. The profile of characteristics of patients with TBI and nTBI causes of injury was also compared.

**Methods:**

A population based retrospective cohort study design with healthcare administrative databases was used. Data on acute care admissions were obtained from the Discharge Abstract Database and patients were identified using the International Classification of Diseases – Version 10 codes for Ontario, Canada from April 1, 2003 to March 31, 2010. Older adults were examined in three age groups – 65 to 74, 75 to 84, and 85+ years.

**Results:**

From 2003/04 to 2009/10, there were 14,518 episodes of acute care associated with a TBI code and 51, 233 episodes with a nTBI code. Overall, the rate of hospitalized TBI and nTBI episodes increased with older age groups. From 2007/08 to 2009/10, the percentage of patients that stayed in acute care for 12 days or more and the percentage of patients with delayed discharge from acute care increased with age. The most common cause of TBI was falls while the most common type of nTBI was brain tumours. The percentage of patients discharged to long term care and complex continuing care increased with age and the percentage discharged home decreased with age. In-hospital mortality also increased with age. Older adults with TBI and nTBI differed significantly in demographic and clinical characteristics and discharge destination from acute care.

**Conclusions:**

This study showed an increased rate of acute care admissions for both TBI and nTBI with age. It also provided additional support for falls prevention strategies to prevent injury leading to cognitive disability with costly human and economic consequences. Implications for increased numbers of people with ABI are discussed.

## Background

Acquired brain injury (ABI) has been defined as damage to the brain that occurs after birth from traumatic or non-traumatic causes [1]. It is a leading cause of death and disability worldwide and the physical, cognitive, psychosocial, and long term consequences of ABI are well documented [2,3]. Recent data from Faul et al. showed that each year, approximately 1.7 million people sustain a traumatic brain injury (TBI) in the United States and it is a contributing factor to 30% of all injury related deaths [4]. Studies have indicated that the rates of TBI are highest among older adults aged 65 years and older. In the United States from 2002 to 2006, the highest rate of TBI was among patients aged 75 years and older (339.3 per 100,000) and the third highest rate was among patients aged 65 to 74 years (113.3 per 100,000) [4]. In Finland from 2001 to 2005, the rates of TBI also increased with age among older adults, with the rate of patients aged 80 years and older the highest at approximately 425 per 100,000 and approximately 210 per 100,000 among patients aged 70 to 79 years [5]. In the province of Ontario in Canada from 1992 to 2002, the highest rates of hospitalizations associated with TBI among both males and females occurred in the 86+ years age group, followed by patients aged 76 to 85 years, and patients aged 66 to 75 years [6]. Updated results from Colantonio and colleagues in Ontario showed that the rates of TBI from 2002 to 2007 continued to increase with age among older adults 65 years and older [7]. Despite this high incidence, there are relatively few papers that specifically focus on TBI among older adults from a population based perspective, and none to date in Canada.

Furthermore, there is an existing paucity of population based research on non-traumatic brain injury (nTBI) outcomes as a whole, which includes diagnoses of non-degenerative conditions such as brain tumours, anoxia, infections and toxic effects among older adults. A recent report by Cancer Care Ontario showed that brain and central nervous system cancer incidence rates in Ontario from 1998 to 2007 peaked at approximately 70 years of age [8]. However, it is important to note that this report did not track benign tumours of the brain and CNS, which may not have the same implications for life expectancy but may still seriously affect cognitive and other functional abilities. An increasing incidence of brain tumours has been reported since the early 1990s. For example, in 1991 Mao et al. found that brain cancer rates among Canadians aged 65 years or older increased by up to 733% from 1969 to 1985 and the rate of hospital admissions among this age group increased by 269% among males and 408% among females [9]. More recently, Arora et al. reported in 2010 an increase in the incidence of primary central nervous system tumours among the elderly in England, with increases of up to 176% for those aged 80 to 84 years [10]. Finally, Lonn et al. in 2004 found an increase in the incidence of brain tumours in four Nordic countries among those aged 60 years and older [11]. There is little population based data providing a detailed examination of these older adults, who now have had better survival rates than ever before.

Despite having very different causes, patients with nTBI often have similar functional sequelae as patients with TBI and are often treated in similar post-acute settings for rehabilitation. However, less is known about this group of patients at risk for long term cognitive disability. A study comparing TBI and nTBI patients in inpatient rehabilitation in Ontario supports a differential profile of patients by type of brain injury [12]. Specifically, it was found that TBI patients were significantly more likely to be male, younger, live in rural areas, and to have had longer lengths of stay in inpatient rehabilitation. Conversely, nTBI patients had significantly more comorbidities and had a higher percentage of mental health diagnoses. However, the extent to which the outcomes of older adults are similar such as in acute care settings is currently not known, as most studies to date have focused on differences across age groups [13,14]. In a recent study by Chen et al. on ABI patients aged 19 years and older discharged from acute care, older adults aged 65 years and over were at least 1.22 times as likely as patients aged 35 to 44 years to be discharged to inpatient rehabilitation compared to home and at least 2.73 times as likely as to be discharged to other institutionalized care compared to home [15]. However, this and other studies [16] do not describe the full range of outcomes (e.g., deaths, discharge to complex continuing care), and thus additional research is required to better understand the outcomes among the nTBI population, and particularly in older adults. A more detailed examination is warranted given the increasing number of older adults at risk for both types of brain injury and their utilization of health care resources.

Currently, there are few population based studies on older adults with TBI and even fewer that specifically examine older adults with nTBI [6,7,12,15,16]. This present study on older adults examined both TBI and nTBI in acute care from a publicly funded healthcare system that is less likely affected by differential access to health care. This study addresses the gaps in research on older adults with ABI. We sought to describe the trends in hospitalization among older adults with ABI as well as their characteristics. Mechanisms of injury were explored and discharge destinations from acute care were identified. Finally, this paper compared TBI and nTBI patients to determine differences among these diagnostic groupings. As of July 1, 2011, Ontario is home to 39% of all Canadians, 14% of which are older adults [17,18]. It is estimated that by the year 2036, older adults will make up a quarter of Canada’s population [19]. As such, it is crucial to identify the trends of ABI among older adults and the characteristics of these patients in order to inform preparation of service delivery for this growing population.

## Methods

### Data source and case definition

The Ontario ABI Dataset was used for this study. Data on all hospitalizations in Ontario were obtained from the Discharge Abstract Database (DAD) from the Ministry of Health and Long-Term Care (MOHLTC). The DAD contains all acute care hospital admissions since 1963 from over 194 publicly funded hospitals in Ontario [20]. Each record in the dataset includes demographic and clinical information about all hospital admissions and discharges, including transfers and deaths, using standard diagnosis and procedure/intervention codes. All hospitals in Ontario are required to submit demographic and clinical information about all hospital admissions and discharges, including transfers and deaths, to the Canadian Institute for Health Information, which collates these data. Trained hospital medical records staff transcribe information from each patient’s medical chart using standard diagnosis (ICD-9, the International Classification of Diseases - 9th revision and ICD-10-CA, the enhanced Canadian version of the 10th revision of the International Statistical Classification of Diseases and Related Health Problems) and procedure/intervention codes (CCP, the Canadian Classification of Procedures and CCI, the Canadian Classification of Health Interventions). Data quality in the DAD has been assessed using chart reabstraction and indicated good agreement for non-clinical variables, moderate to substantial agreement for the most responsible diagnoses, and good specificity of ABI codes [21]. Residents of Ontario have universal access to hospital-based care.

ABI cases were identified in the DAD by the presence of an International Classification of Diseases – Version 10 (ICD-10) code for TBI and nTBI in any diagnosis position (up to 25, including primary and secondary diagnoses). The ICD-10 codes used were based on a literature review and stakeholder consultation in the Canadian context [22]. TBI codes were categorized into three diagnoses – fracture and crushing of the skull and facial bones (S02.0, S02.1, S02.3, S02.7, S02.8, S02.9, S07.1), intracranial injury, excluding those with skull fracture (S06.0, S06.1, S06.2, S06.3, S06.4, S06.5, S06.6, S06.7, S06.8, S06.9), and late effects of injuries (F07.2, T90.2, T90.5). NTBI included anoxia (G93.1, T71, T75.1, R09.0), brain tumours (C70, C71, C79.3, C79.4, D32.0, D33.0, D33.1, D33.2, D33.3, D42.0, D43, D43.2, G06.0, G06.1, G06.2, G07, G93.0), encephalitis (A81.1, A83.0, A83.2, A86.0, B00.4, B01.1, B02.0, B05.0, B94.1, G04.0, G04.2, G04.8, G04.9, G05, G09), meningitis (A87, B01.0, B37.5, G00, G01, G02, G03), metabolic encephalopathy (E10.0, E11.0, E13.0, E14.0, E15, G92, G93.4), other brain disorders and infections (G91.0, G91.1, G91.2, G93.2, G93.5, G93.6, G93.8, G93.9, G99.8, R29.1), toxic effects of substances, chiefly non-medical as to source (T40.5, T42.6, T51, T56, T57.0, T57.2, T57.3, T58, T64, T65.0), and vascular insults not captured in other national studies of stroke (I62.0, I62.9). We excluded stroke patients in the nTBI group when it was in the most responsible diagnosis field and anywhere in TBI diagnosis fields.

### Variables

Rates were calculated using the number of unique ABI episodes divided by the population intercensal estimates, obtained from Statistics Canada, expressed per 100,000 persons. Demographic variables included age and sex. Older adults were defined as 65 years and older and were categorized in three different age groups (65 – 74, 75 – 84, and 85+ years). Older adults aged 65 years and older were examined as one group when comparing TBI and nTBI patients. Fiscal years 2003/04 to 2009/10 were examined to determine trends in the rates of ABI hospitalizations over time, while fiscal years 2007/08 to 2009/10 were examined to determine the characteristics and mechanisms of injury in acute care during this time period.

Clinical variables included the Charlson Comorbidity Index, length of stay in acute care, number of alternate level of care days, and number of special care days. Comorbidities were analyzed using the Charlson Comorbidity Index, categorized into scores of 0 – 1 (low), 2 – 3, and 4+ (high) [23]. This index is widely accepted as a useful tool for measuring comorbidity disease status and has shown to have a consistent correlation to in-hospital mortality [24]. Each Charlson Comorbidity was also examined. For all DAD records, all multiple Charlson Comorbidities were included. Length of stay (LOS) in acute care was defined as the number of days between admission and discharge in acute care. Alternate level of care (ALC) days is a quality of care indicator that represents hospital beds occupied by patients who do not need the intensity of acute care services and who would be more appropriately cared for in other settings [25]. ALC days were calculated as the sum of all days in ALC. Special care days were defined as the cumulative number of days spent in all intensive care units. Discharge disposition from acute care included death in acute care, home, inpatient rehabilitation, complex continuing care (CCC), long term care (LTC), and 'other’. This was measured using an algorithm between 2 variables, discharge disposition and institutional transfer type.

Mechanism of injury variables were classified according to the Centers for Disease Control and Prevention (CDC) external cause of injury matrix [26]. These variables included falls, motor vehicle collision (MVC), struck by/against, and other. Falls were further categorized into falls on the same level (W00, W01, W03, W18), falls from one level to another (W11, W12, W13, W14, W15, W16, W17), falls involving furniture (W06, W07, W08), falls involving wheelchair or walker (W05, W0501), falls on and from stairs and steps (W10), and other falls and unspecified falls (W0502, W0503, W02, W0201, W0203, W0204, W0208, W04, W09, W0901, W0902, W0904, W0905, W0908, W0909, W0508, W0509, Y30, W19). For each DAD record, all multiple external causes of injury codes were included. The types of nTBI were also examined by age groups to determine the cause of nTBI among older adults. For each DAD record, all multiple nTBI codes were included.

### Analyses

TBI and nTBI patient data were analyzed separately. All hospital separations were grouped into episodes of care using a 24 hour rule, such that admissions that occurred within 24 hours of a previous discharge were considered part of the same episode of care. Only the last hospital separation was considered. Chi-square tests were conducted to examine (1) the association between age categories and characteristics, mechanism/type of injury, and discharge destination, and (2) whether older adults with TBI and nTBI differ in select demographic and clinical characteristics and in their discharge destinations from acute care. Note that due to the large sample size, even small differences were statistically significant. Univariate odds ratios were produced for comparisons between TBI and nTBI patients. For the patient characteristic tables only, the first ABI episode per patient was used; therefore, this is a patient level analysis (vs. hospitalization episode level). Bonferroni correction was applied, with a criterion significance of p < .05 divided by the number of comparisons conducted.

### Privacy and ethics

Research ethics approval was received from the Toronto Rehabilitation Institute Research Ethics Board. All investigators and staff involved in the study signed confidentiality agreements and analyses were conducted with de-identified data.

## Results

### Traumatic brain injury

From fiscal years 2003/04 to 2009/10, there were 14,518 TBI hospitalization episodes among older adults. During this period, the rate of TBI increased in all three age groups. Specifically, the rate of hospitalized TBI episodes among patients 65 to 74 years of age increased by 11%, the rate among patients aged 75 to 84 years increased by almost 50%, and the rate among patients aged 85 years and older increased by 63%. Each year, the rate of hospitalized TBI episodes in the 85 years and older age group was also higher than the 75 to 84 and 65 to 74 year age groups. In 2009, the rate of patients aged 85 years and older (361 per 100,000) was 1.89 the times the rate of patients aged 75 to 84 years (191 per 100,000) and was 4.63 times the rate of patients aged 65 to 74 years (78 per 100,000) (see Table 1).

**Table 1:** Number and rate of hospitalized ABI episodes by age groups in Ontario, 2003/04 – 2009/10

**Fiscal year of discharge**	**Age groups**					
	**65 – 74 Years**		**75 – 84 Years**		**85+ Years**	
	**N**	**Rate per 100,000**	**N**	**Rate per 100,000**	**N**	**Rate per 100,000**
**TBI**						
**2003/04 – 2009/10**	4,348	71	6,294	153	3,876	286
**2003/04**	589	70	705	128	361	222
**2004/05**	500	59	761	134	440	262
**2005/06**	549	64	836	144	436	243
**2006/07**	604	70	866	147	547	283
**2007/08**	679	76	964	160	563	272
**2008/09**	698	76	1,009	166	720	326
**2009/10**	729	78	1,153	191	809	361
**nTBI**						
**2003/04 – 2009/10**	22,840	372	20,884	509	7,509	554
**2003/04**	3,360	400	3,091	562	990	610
**2004/05**	3,297	388	3,050	538	1,060	630
**2005/06**	3,337	390	3,160	545	1,095	611
**2006/07**	3,126	360	2,827	478	1,035	535
**2007/08**	3,166	357	2,775	462	990	478
**2008/09**	3,161	346	2,945	483	1,081	490
**2009/10**	3,393	365	3,036	502	1,258	561

Note: This is an episode level analysis.

### Mechanism of injury

Falls were the leading cause of TBI among hospitalized older adults, followed by MVC. According to the chi-square test, the association of mechanism of injury and age categories was statistically significant (p < .001). As the age groups increased, the percentage of TBI due to falls increased from 70% to 88% while the percentage of TBI due to MVC decreased from 11% to 4%. Among patients who fell, the most common types of falls were falls on the same level and falls on and from stairs and steps. With increasing age, the percentage of older adults that fell on the same level increased from 34% to 41% while the percentage that fell on and from stairs and steps decreased from 21% to 12% (see Table 2).

**Table 2:** Mechanism of injury among TBI patients by age groups in Ontario, 2007/08 – 2009/10

Mechanism of injury ***	**Age groups**					
	**65 – 74 Years**		**75 – 84 Years**		**85+ Years**	
	**N**	**Col%**	**N**	**Col%**	**N**	**Col%**
**Total**	2,308	100	3,398	100	2,201	100
**Fall**	1,610	69.8	2,752	81.0	1,932	87.8
On the same level	543	33.7	1087	39.5	782	40.5
From one level to another	164	10.2	97	3.5	27	1.4
Involving furniture	66	4.1	126	4.6	135	7.0
Involving wheelchair/walker	25	1.6	28	1.0	68	3.5
On and from stairs and steps	339	21.1	445	16.2	236	12.2
Other and unspecified	473	29.4	969	35.2	684	35.4
**Motor vehicle collision**	261	11.3	219	6.4	77	3.5
**Struck by/against**	80	3.5	68	2.0	34	1.5
**Other**	271	11.7	258	7.6	96	4.4
**Missing**	86	3.7	101	3.0	62	2.8

Statistical Significance (chi-square): *** p < .001.

Note: This is a record level analysis and includes all records with an ABI diagnostic code, as defined in the Methods section.

### Demographic and clinical characteristics

From fiscal years 2007/08 to 2009/10, 29% of all hospitalized older adults with TBI were 85 years and older. Statistical differences among the 3 age groups were found for sex (p < .001), Charlson Comorbidity Index score (p < .05), LOS in acute care (p < .001), ALC days (p < .001), and special care days (p < .001). However, after Bonferroni correction, Charlson Comorbidity Index score was not significant. The majority were male (54%), however, with increasing age, the percentage of males decreased from 64% to 44% while the percentage of females increased from 36% to 57%. During this period, 21% of TBI patients had a Charlson Comorbidity Index score of 2 or higher, 37% stayed in acute care for 12 days or longer, 23% had at least one ALC day, and 26% had at least one special care day. As the age groups increased, the percentage of patients that stayed in acute care for 12 days or more increased from 33% to 41% and the percentage with at least one ALC day increased from 18% to 30%. Conversely, the percentage of cases with at least one special care day decreased from 34% to 17% (see Table 3). The top three types of Charlson Comorbidities among older adults with TBI were diabetes with no organ failure (17%), dementia (11%), and diabetes with organ failure (7%) (see Table 4).

**Table 3:** Characteristics of hospitalized TBI patients by age groups in Ontario, 2007/08 to 2009/10

**Characteristics**	**Age groups**							
	**Older adults (65+ Years)**		**65 – 74 Years**		**75 – 84 Years**		**85+ Years**	
	**N**	**Col %**	**N**	**Col %**	**N**	**Col %**	**N**	**Col %**
Overall	6,511	100	1,868	100	2,759	100	1,884	100
Sex ***								
Males	3,482	53.5	1,189	63.7	1,474	53.4	819	43.5
Females	3,029	46.5	679	36.3	1,285	46.6	1,065	56.5
^**1 **^Charlson comorbidity index *								
0 – 1 (low)	5,169	79.4	1,518	81.3	2,155	78.1	1,496	79.4
2 – 3	1,043	16.0	261	14.0	468	17.0	314	16.7
4+ (high)	299	4.6	89	4.8	136	4.9	74	3.9
Length of stay in acute care (days) ***								
1 – 2	824	12.7	246	13.2	345	12.5	233	12.4
3 – 5	1,602	24.6	501	26.8	696	25.2	405	21.5
6 – 11	1,679	25.8	505	27.0	694	25.2	480	25.5
12+	2,406	37.0	616	33.0	1,024	37.1	766	40.7
Alternate level of care days ***								
None	5,018	77.1	1,528	81.8	2,163	78.4	1,327	70.4
1 – 2	180	2.8	43	2.3	73	2.6	64	3.4
3 – 5	240	3.7	69	3.7	93	3.4	78	4.1
6 – 11	361	5.5	81	4.3	145	5.3	135	7.2
12+	712	10.9	147	7.9	285	10.3	280	14.9
Special care days ***								
None	4,810	73.9	1,237	66.2	2,016	73.1	1,557	82.6
1 – 2	657	10.1	226	12.1	300	10.9	131	7.0
3 – 5	461	7.1	179	9.6	184	6.7	98	5.2
6 – 11	304	4.7	103	5.5	146	5.3	55	2.9
12+	279	4.3	123	6.6	113	4.1	43	2.3
Discharge destination ***								
Death	1,344	20.6	255	13.7	553	20.0	536	28.5
Home	3,087	47.4	1,096	58.7	1,315	47.7	676	35.9
Inpatient rehabilitation	708	10.9	235	12.6	329	11.9	144	7.6
Complex continuing care	360	5.5	90	4.8	141	5.1	129	6.8
Long term care	606	9.3	65	3.5	236	8.6	305	16.2
Other	406	6.2	127	6.8	185	6.7	94	5.0

Statistical Significance (chi-square): * p < .05; ** p < .01; *** p < .001.

This is a patient level analysis. Note: ^1^After Bonferroni correction, Charlson Comorbidity Index score was not significant.

**Table 4:** Prevalence of diagnoses captured by the Charlson comorbidity index by type of brain injury in Ontario, 2007/08 – 2009/10

Diagnosis***	**TBI N (%)**	**nTBI N (%)**
**Overall**	6,614 (100)	17 794 (100)
**Metastatic cancer**	93 (1.4)	4,928 (27.7)
**Cerebral vascular disease**	308 (4.7)	3,662 (20.6)
**Diabetes with no organ failure**	1,133 (17.1)	3,336 (18.8)
**Primary cancer**	190 (2.9)	2,386 (13.4)
**COPD/other respiratory disorder**	302 (4.6)	2,196 (12.3)
**Diabetes with organ failure**	453 (6.9)	1,899 (10.7)
**Congestive heart failure**	321 (4.9)	1,486 (8.4)
**Dementia**	734 (11.1)	1,329 (7.5)
**Acute myocardial infarction**	236 (3.6)	1,261 (7.1)
**Renal disease**	273 (4.1)	1,112 (6.3)
**Hemiplegia**	192 (2.9)	650 (3.7)
**Peripheral vascular disease**	67 (1.0)	329 (1.9)
**Rheumatologic disease**	41 (0.6)	147 (0.8)
**Mild liver disease**	15 (0.2)	129 (0.7)
**Digestive ulcer**	16 (0.2)	112 (0.6)
**HIV infection**	0	5 (0.03)

Note: This is a record level analysis and includes only records with a Charlson Comorbidity. For each DAD record, all multiple Charlson Comorbidities were included. Statistical significance (chi-square): *** p < .001.

### Discharge destination

Chi-square tests showed a significant association between age categories and discharge destination (p < .001). Overall, almost 50% of older adults were discharged home after acute care, followed by inpatient rehabilitation (11%) and LTC (9%). Approximately one fifth of older adults died in acute care. The percentage of older adults discharged home and to inpatient rehabilitation decreased from 59% to 36% and 13% to 8% respectively with increasing age; the percentage of patients discharged to CCC and LTC increased from 5% to 7% and from 4% to 16% respectively. With increasing age, the percentage of older adults that died in acute care increased from 14% to 29% (see Table 3).

### Non-traumatic brain injury

From fiscal years 2003/04 to 2009/10, there were 51,233 nTBI hospitalization episodes among older adults. During this period, there was an overall decreasing trend in the rate of hospitalized nTBI episodes; however, it fluctuated between 2003/04 and 2009/10. From 2003/04 to 2009/10, the rate of hospitalized nTBI episodes among patients 65 to 74 years of age decreased by 9%, the rate among patients aged 75 to 84 years decreased by 11%, and the rate among patients aged 85 years and older decreased by 8%. However, from 2007/08 to 2009/10, there was a 2% increase in the rate of hospitalized nTBI episodes among patients aged 65 to 74 years, 9% among patients aged 75 to 84 years, and 17% among patients aged 85+ years. With older age groups, the rate of hospitalized nTBI episodes from 2003/04 to 2009/10 also increased. In 2009/10, the rate of hospitalized nTBI episodes among patients aged 85 years and older in acute care (561 per 100,000) was 1.12 times the rate of patients aged 75 to 84 years (502 per 100,000), which was 1.38 times the rate of patients aged 65 to 74 years (365 per 100,000) (see Table 1).

### Type of Non-traumatic brain injury

Chi-square tests revealed a significant association between age categories and type of nTBI (p < .001). The most common types of nTBI among hospitalized older adults from fiscal years 2003/04 to 2009/10 were brain tumours (44%), anoxia (20%), and vascular insults (14%). With increasing age the percentage of nTBI due to brain tumours decreased from 53% among patients aged 65 to 74 years to 26% among patients aged 85 years and older. Conversely, the percentage due to anoxia and vascular insults increased from 16% to 28% and from 9% to 23% respectively (see Table 5).

**Table 5:** Type of nTBI by age groups in Ontario, 2003/04 – 2009/10

Cause of Injury ***	**Age groups**							
	**Older adults (65+ Years)**		**65 – 74 Years**		**75 – 84 Years**		**85+ Years**	
	**N**	**Col%**	**N**	**Col%**	**N**	**Col%**	**N**	**Col%**
**Total**	58,628	100	26,484	100	23,917	100	8,227	100
**Anoxia**	11,883	20.3	4,347	16.4	5,210	21.8	2,326	28.3
**Brain Tumour**	25,727	43.9	14,073	53.1	9,510	39.8	2,144	26.1
**Encephalitis**	971	1.7	480	1.8	359	1.5	132	1.6
**Meningitis**	1,052	1.8	558	2.1	373	1.6	121	1.5
**Metabolic encephalopathy**	4,839	8.3	1,859	7.0	2,116	8.8	864	10.5
**Other brain disorders and infections**	5,301	9.0	2,309	8.7	2,310	9.7	682	8.3
**Toxic effects**	841	1.4	463	1.7	283	1.2	95	1.2
**Vascular insults**	8,014	13.7	2,395	9.0	3,756	15.7	1,863	22.6

Note: This is a record level analysis. For each DAD record, all multiple nTBI codes were included.

Statistical significance (chi-square): *** p < .001.

### Demographic and clinical characteristics

From fiscal years 2007/08 to 2009/10, 17% of all hospitalized older adults with nTBI were 85 years or older. Statistical differences among the 3 age groups were found for sex (p < .001), Charlson Comorbidity Index score (p < .001), LOS in acute care (p < .001), ALC days (p < .001), and special care days (p < .001). While there was a slightly higher percentage of males overall (52%), with increasing age, the percentage of females increased from 45% to 57% in the highest age group. During this period, 62% had a Charlson Comorbidity Index score of 2 or higher, 43% stayed in acute care for 12 days or longer, 19% had at least one ALC day, and 28% had at least one special care day. With older age, the percentage of patients that stayed in acute care for 12 days or more increased from 40% to 43% and the percentage with at least one ALC day increased from 14% to 24%. Conversely, the percentage of cases with at least one special care day decreased from 32% to 18% and the percentage with a Charlson Comorbidity Index score of 2 or more decreased from 66% to 54% (see Table 6). The top three types of Charlson Comorbidities among older adults with nTBI were metastatic cancer (28%), cerebral vascular disease (21%), and diabetes with no organ failure (19%) (see Table 4).

**Table 6:** Characteristics of hospitalized nTBI patients by age groups in Ontario, 2007/08 to 2009/10

**Characteristics**	**Age groups**							
	**Older adults (65+ Years)**		**65 – 74 Years**		**75 – 84 Years**		**85+ Years**	
	**N**	**Col %**	**N**	**Col %**	**N**	**Col %**	**N**	**Col %**
Overall	16,958	100	7,092	100.0	6,967	100.0	2,899	100.0
Sex ***								
Males	8,826	52.0	3,905	55.1	3,683	52.9	1,238	42.7
Females	8,132	48.0	3,187	44.9	3,284	47.1	1,661	57.3
Charlson comorbidity index ***								
0 – 1 (low)	6,445	38.0	2,416	34.1	2,689	38.6	1,340	46.2
2 – 3	4,661	27.5	1,769	24.9	2,016	28.9	876	30.2
4+ (high)	5,852	34.5	2,907	41.0	2,262	32.5	683	23.6
Length of stay in acute care (days) ***								
1 – 2	1,675	9.9	677	9.5	690	9.9	308	10.6
3 – 5	3,349	19.7	1,567	22.1	1,255	18.0	527	18.2
6 – 11	4,730	27.9	2,016	28.4	1,905	27.3	809	27.9
12+	7,204	42.5	2,832	39.9	3,117	44.7	1,255	43.3
Alternate level of care days ***								
None	13,777	81.2	6,082	85.8	5,499	78.9	2,196	75.8
1 – 2	455	2.7	154	2.2	195	2.8	106	3.7
3 – 5	520	3.1	173	2.4	223	3.2	124	4.3
6 – 11	797	4.7	247	3.5	371	5.3	179	6.2
12+	1,409	8.3	436	6.1	679	9.7	294	10.1
Special care days ***								
None	12,176	71.8	4,810	67.8	4,999	71.8	2,367	81.6
1 – 2	1,830	10.8	909	12.8	708	10.2	213	7.3
3 – 5	1,187	7.0	546	7.7	492	7.1	149	5.1
6 – 11	923	5.4	425	6.0	403	5.8	95	3.3
12+	842	5.0	402	5.7	365	5.2	75	2.6
Discharge destination ***								
Death	5,129	30.2	1,949	27.5	2,151	30.9	1,029	35.5
Home	7,512	44.3	3,697	52.1	2,903	41.7	912	31.5
Inpatient rehabilitation	1,002	5.9	396	5.6	446	6.4	160	5.5
Complex continuing care	1,229	7.2	397	5.6	556	8.0	276	9.5
Long term care	1,082	6.4	186	2.6	502	7.2	394	13.6
Other	1,004	5.9	467	6.6	409	5.9	128	4.4

Statistical Significance (chi-square): *** p < .001.

Note: This is a patient level analysis.

### Discharge destination

Chi-square tests showed a significant association between age categories and discharge destination (p < .001). Overall, from fiscal years 2007/08 to 2009/10, 44% were discharged home from acute care, followed by CCC (7%), LTC (6%), and inpatient rehabilitation (6%). Almost one third of nTBI patients in acute care died. As the age groups increased, the percentage of patients discharged home decreased from 52% to 32% while percentage to CCC and LTC increased from 6% to 10% and from 3% to 14% respectively. The percentage discharged to inpatient rehabilitation remained relatively stable throughout the age groups. With increasing age, the percentage of older adults with nTBI that died in acute care increased from 28% to 36% (see Table 6).

### Traumatic vs. Non-traumatic brain injury

#### Demographic and clinical characteristics

Chi-square tests revealed a statistically significant association between the type of brain injury and specific demographic and clinical characteristics. A significantly higher proportion of older adults with nTBI were aged 65 to 74 years (p < .001) while a significantly higher proportion of older adults with TBI were aged 85+ years. A significantly higher proportion of older adults with nTBI had a Charlson Comorbidity Index score of 2 or higher (p < .001), stayed in acute care for 12 days or longer (p < .001), and had at least one special care day (p < .01). Univariate odds ratio showed that nTBI patients were 6.28 times more likely to have a Charlson Comorbidity Index score of 2 or higher, 1.26 times more likely to stay in acute care for 12 days or longer, and 1.11 times more likely to have at least one special care day. Conversely, TBI patients were 1.29 times more likely to have at least one ALC day. One of the common top three Charlson Comorbidities among both TBI and nTBI patients was diabetes with no organ failure. Chi-square test revealed that a significantly higher proportion of older adults with nTBI had this comorbidity (p < .01).

#### Discharge destination from acute care

Chi-square tests showed a statistically significant association between the type of brain injury and discharge destination from acute care. Specifically, a significantly higher proportion of TBI patients were discharged home (p < .001), to inpatient rehabilitation (p < .001), and to LTC (p < .001). Conversely, significantly more nTBI patients were discharged to CCC (p < .001) and died in acute care (p < .001). Univariate odds ratio showed that TBI patients were 1.13 times more likely to be discharged home, 1.93 times more likely to be discharged to inpatient rehabilitation, and 1.51 times more likely to be discharged to LTC. However, nTBI patients were 1.34 times more likely to be discharged to CCC and 1.67 times more likely to die in acute care.

## Discussion

This paper is the first, to our knowledge, to focus on older adults only with ABI in Ontario, Canada, by older adult age groups. Specifically, this paper examined the trends and profile of hospitalized older adults with TBI and nTBI diagnosis codes with preliminary comparisons between TBI and nTBI patients in this care setting.

From 2003/04 to 2009/10, the rate of TBI in acute care increased while there was an overall decreasing trend in the rate of nTBI. However, each year, the overall rate of ABI increased with age. In 2009/10 alone, the rate of TBI among older adults aged 85+ years was 89% higher than the rate among patients aged 75 to 84 years and 362% higher than the rate among patients aged 65 to 74 years. Similarly, the rate of nTBI among older adults aged 85 years and older was 12% higher than the rate among patients between the ages of 75 to 84 years and 54% higher than the rate among patients aged 65 to 74 years.

The finding that the rate of nTBI fluctuated from 2003/04 to 2009/10 is in line with recent cancer statistics from the United States [27]. Periods of increasing rates of nTBI may be due to improved diagnostic techniques, which may result in more cases of nTBI detected. Studies from a range of countries have found increasing rates of brain tumours among older adults and have cited increased availability of neuroimaging techniques as potentially responsible for the increase in diagnoses. Better and more accessible diagnostic imaging may also result in more care offered in outpatient settings versus in hospital [9-11]. The finding that the rate of TBI increased from 2003/04 to 2009/10 corroborates recent findings from other developed countries like Australia, where Harvey and Close examined TBI cases in the hospital from 1998 to 2011. They found that during this period, the hospitalization rate for TBI among older adults increased by 7.2%, with a consistently higher rate among males [28]. Older adults are the fastest growing segment of the population; thus, policy makers need to prepare for an increase of ABI in acute care, especially among older adults, from both prevention and health care delivery perspectives. The focus on cognitive disability among older adults caused by dementia and stroke clearly demonstrates the importance of understanding the distinct profiles of these patients to plan accordingly. For example, dementia and cerebrovascular disease were common in the TBI and nTBI patient samples. Understanding the relationships between these types of brain injury, and ensuring that the effects of different types of brain injury be appropriately assessed and managed, are crucial. We and others have shown that older adults can make significant functional cognitive gains in inpatient rehabilitation [29,30]. Therefore, it is important that services provided in inpatient rehabilitation be offered to older adults with ABI.

The finding that falls are the leading cause of TBI among older adults in acute care aligns with results from around the world [4-6,16,31,32]. The dramatic increase in TBI due to falls illustrates the need for an increased focus on falls prevention strategies among older adults. This study is the first population based study, to our knowledge, to show patterns of falls by subtypes. It showed that age differences exist by type of falls. Specifically, among TBI patients that sustained their injury as a result of a fall, the percentage that fell on the same level increased with age while the percentage that fell on and from stairs and steps decreased. In a 2003 study on blunt head trauma using the Ontario Trauma Registry datasets, Pickett, Simpson, and Brison found that the highest number of falls was among older patients aged 60 years and up. Among this age group, the leading falls subtypes were also falls on the same level and on stairs and steps [33]. However, this current study provides much more detail about the cause of falls by more specific age groups among the older adult population. This is important, as Luukinen and colleagues concluded from a population based study that falls related TBI predicted earlier onset of dementia [34]. Also, a TBI with an existing dementia, for instance, can be an additional risk factor for a fall due to balance and other problems that may result. Reviews on Alzheimer’s disease and TBI have also supported a link between these two conditions [35,36] and studies have shown that having a TBI is a significant predictor of premature mortality [37,38]. As such, detailed examination of the types of falls in this population is critical to inform falls prevention strategies and in particular, to prevent another fall. Persons with TBI may be at risk for subsequent falls due to balance, mobility, and cognitive disability as well as environmental challenges. Preventing re-injury should be a goal and education is emerging regarding falls prevention in this group [39]. It is also acknowledged that factors such as substance use, dementia, and other neurological conditions can also lead to a fall. Future studies should identify significant predictors of falls and falls subtypes among older adults.

Demographic and clinical characteristics of hospitalized ABI patients also differed by age groups. In particular, as the age groups increase, the percentage of female patients with both TBI and nTBI increased. The percentage of patients with nTBI due to anoxia increased while the percentage due to brain tumours and vascular insults decreased with age. Moreover, the percentage of ABI patients that stayed in acute care for 12 days or more and had at least one ALC day also increased with age. This suggests that if the trends in the rate of TBI continue, policy makers and health care professionals need to prepare for the increase in the length of stay and more delays in discharge from acute care in the coming years as the proportion of older adults increases in Ontario. This is particularly important, as ALC days are very costly to the health care system and are not desirable in terms of the patient experience. Further, while TBI overall is more common in males, the sex distribution among this older hospitalized cohort is fairly equitable. As such, it is important to address sex specific issues in both TBI and nTBI populations.

Findings on discharge destination from acute care showed that among both TBI and nTBI patients, the percentage that were discharged to CCC and LTC increased with age. Unpublished observations from Colantonio and colleagues found that approximately 50% of patients in the CCC with TBI are older adults. This suggests that there is a demand for the use of these health care services in the near future without any other options in place. Moreover, given that the percentage of patients discharged home decreased with age, providing enhanced home services that meet the patients’ needs should be explored in an attempt to discharge more patients home to age in place. Future research examining discharge disposition should examine the category of home in more detail. For instance, examining discharges of persons who went home with and without support services may reveal differences among these patients. Specifically, patients discharged home with home services may have more health problems. However, it may also identify whether individuals received needed supports, and therefore assist in the planning of health care services in the community. For example, in an adult population of ABI patients aged 19 years and older, Chen et al. in 2012 found that older adults were significantly more likely to be discharged to inpatient rehabilitation or institutionalized care after acute care [15]. Research examining the predictors of discharge to various health care settings and of death exclusively among older adults that controls for significant confounders such as age, however, is needed.

Finally, this paper suggests that older adults with TBI and nTBI are distinct populations, with significantly more nTBI patients with a Charlson Comorbidity Index score of 2 or higher, staying in acute care for 12 days or longer, and with one or more special care days. Discharge destinations also differed significantly by type of brain injury, with more TBI patients discharged home, to inpatient rehabilitation, and to LTC after acute care. The difference in percentage discharged to rehabilitation was most notable among these discharge destinations and could explain why more TBI patients had more ALC days as they may be waiting for inpatient rehabilitation services versus being discharged elsewhere. Of particular importance is that significantly more nTBI patients had a Charlson Comorbidity Index score of 2 or higher, with odds ratio indicating that these patients were 6.28 times more likely than TBI patients to have this score. The high Charlson Comorbidity Index score could also explain the higher percentage of nTBI patients with in-hospital mortality and the type of diagnoses such as brain tumours, which may have a poorer prognosis. A previous study by Colantonio et al. also demonstrated that nTBI patients in inpatient rehabilitation across all age groups had significantly more comorbidities and had a significantly higher percentage of mental health diagnoses [12]. Thus, this finding indicates that in both health care settings, comorbidities are a prevalent issue in the ABI population, especially among nTBI patients. This finding is of particular importance, as research on TBI patients with comorbidities has indicated that comorbidities have an adverse impact on the outcome of these patients [40-42]. Moreover, this finding suggests that health care professionals must be prepared for the existence of comorbidities among ABI patients and in particular, older adults with nTBI. This paper provided the basis for future comparisons between older adults with TBI and nTBI, which is important, given that these patients are often placed side by side in the rehabilitation setting. Understanding the differences in the profiles and outcomes of these two populations may lead to more targeted rehabilitation programs for these patients.

### Strengths and limitations

The strength of this project is that the Ontario ABI Dataset captures information on both traumatic and non-traumatic brain injury across the entire provincial population and the definition of ABI in this dataset covers mild TBI as well. While many registries exist that examine TBI internationally, there are little population based data on brain injury from non-traumatic causes even though persons with nTBI may have similar functional disabilities despite the varying mechanisms of injury. Also, data on hospitalizations were obtained from the DAD, which contains all acute care hospitalizations in the province of Ontario [20]. Ontario has publicly funded health care and therefore the data may have less bias as a result of non-differential access to care. Ontario accounts for 40% of all Canadians [17], thus findings from this study are highly generalizable and can inform other provinces in Canada.

However, limitations associated with the use of administrative data must be recognized. First, information on discharge destination was not based on actual linkage of records across the continuum of care; thus, misclassification bias is possible. Second, patients were identified if they had an ABI code in any diagnosis position, regardless of whether the brain injury was the most responsible diagnosis during the study period and thus, the population under study may differ with regard to their brain injuries. As such, a brain injury may not have been the most responsible diagnosis (i.e., the condition most responsible for their length of stay in acute care) in the records included in this study. Additionally, while not a limitation to the study, it should be noted that we also used a relatively conservative definition of TBI compared to other studies [4] so comparisons should be made with this in mind. Furthermore, there is currently no international consensus of what constitutes nTBI. We propose more discussion regarding consensus of diagnostic classifications regarding nTBI. This is important, given that we expect higher survival rates in the future with implications for improved quality of life.

## Conclusions

This paper provides a compelling argument for giving more attention to older adults with ABI. With the aging population and the increase in the rate of ABI in acute care as people age, a focus on prevention strategies targeted at the older adult population is crucial. Falls are a major cause of TBI and injuries to the head should be assessed when a fall occurs. Moreover, health care services need to prepare for an increasing number of older adults with potential long term cognitive and other impairments accessing services such as LTC and CCC. Clinicians serving older adults with acquired brain injury should be educated about the best way to screen, diagnose and manage these potentially life long conditions. The need for long term community support for these individuals and their families to avert more costly care is an important policy issue. Research into how specific patient characteristics and, in particular, comorbidities among this vulnerable population will assist in the planning and improvement of services for ABI patients.

## Competing interests

The authors declare that they have no competing interests.

## Authors’ contributions

VC, AC, BZ, and DP conceptualized the study. BZ and VC formulated the methods for statistical analysis and carried out the statistical analysis using SAS software. VC drafted the paper, conducted the literature review, and interpreted the results that formulated the foundation of the paper. VC, AC, BZ, and DP all had significant input into the editing process of the paper and additional interpretation of results. All authors read and approved the final manuscript.

## Pre-publication history

The pre-publication history for this paper can be accessed here:

http://www.biomedcentral.com/1471-2318/13/97/prepub
